# Supplementary material for: Delayed formation of neural representations of space in aged mice
Source: Aging Cell. 2023 Jul 25;22(9):e13924. doi: 10.1111/acel.13924 (PMC10497831; doi:10.1111/acel.13924)
Supplement: Supplementary file 1 — Appendix S1. [file ACEL-22-e13924-s001.zip › acel13924-sup-0001-AppendixS1.pdf]

Figure S1. Velocity and laps run during two photon imaging. A) Average velocity of mice walking along treadmill belt. B) Average number of laps run per imaging session. Statistics done with mixed effects model. Each point represent an individual animal. Bars represent mean +/- SEM. ns=not significant.

Figure S2. No difference in active cell number between young and aged mice. A) Percentage of cells active out of the total cells in a field of view, by mouse. Statistics done with mixed effects model. Bars represent mean +/- SEM. ns=not significant. B) Correlation of percentage active cells versus the total number of cells pooled from all imaging days. Data are shown by mouse.

Figure S3. Aged mice show a deficit in a spatial memory task. A) Schematic of the object placement behavioral paradigm. B) Novelty preference of mice measured by percentage of total time spent exploring the novel object. C) Discrimination index calculated by dividing the difference in exploration time between objects by the total exploration time [(novel-familiar)/(novel+familiar)]. D) Exploration time dedicated to each object. Statistics in B and C were done with one sample Wilcoxon tests; D with mixed effects model. Bars represent mean +/- SEM. Young N=13, Aged N=13. ns=not significant, *p<0.05, **p<0.01.

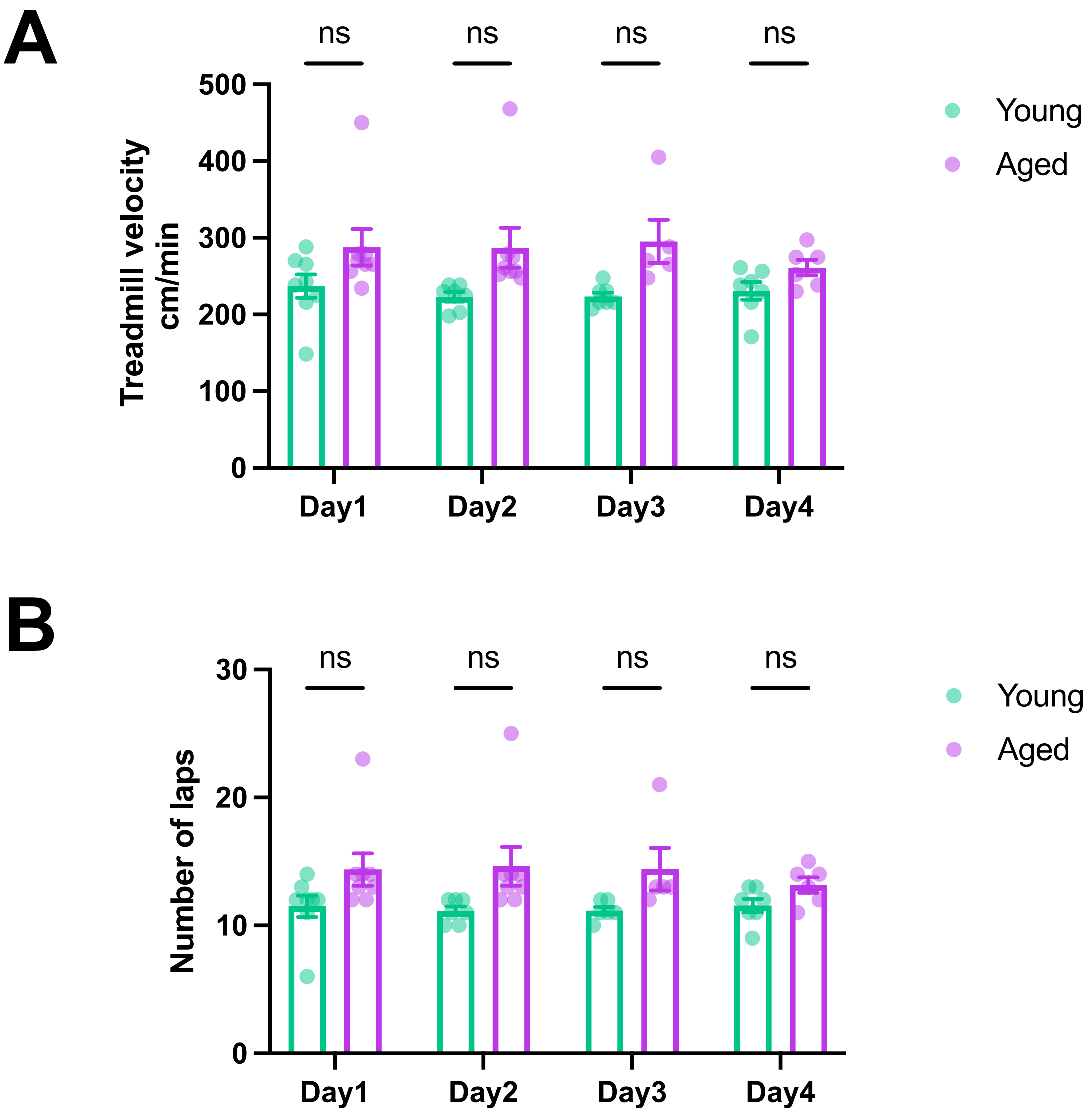
A
ns
ns
ns
ns
Treadmill velocity cm/min
500
400
300
200
100
0
Day1
Day2
Day3
Day4
Young
Aged
B
ns
ns
ns
ns
Number of laps
30
20
10
0
Day1
Day2
Day3
Day4
Young
Aged

Figure S1

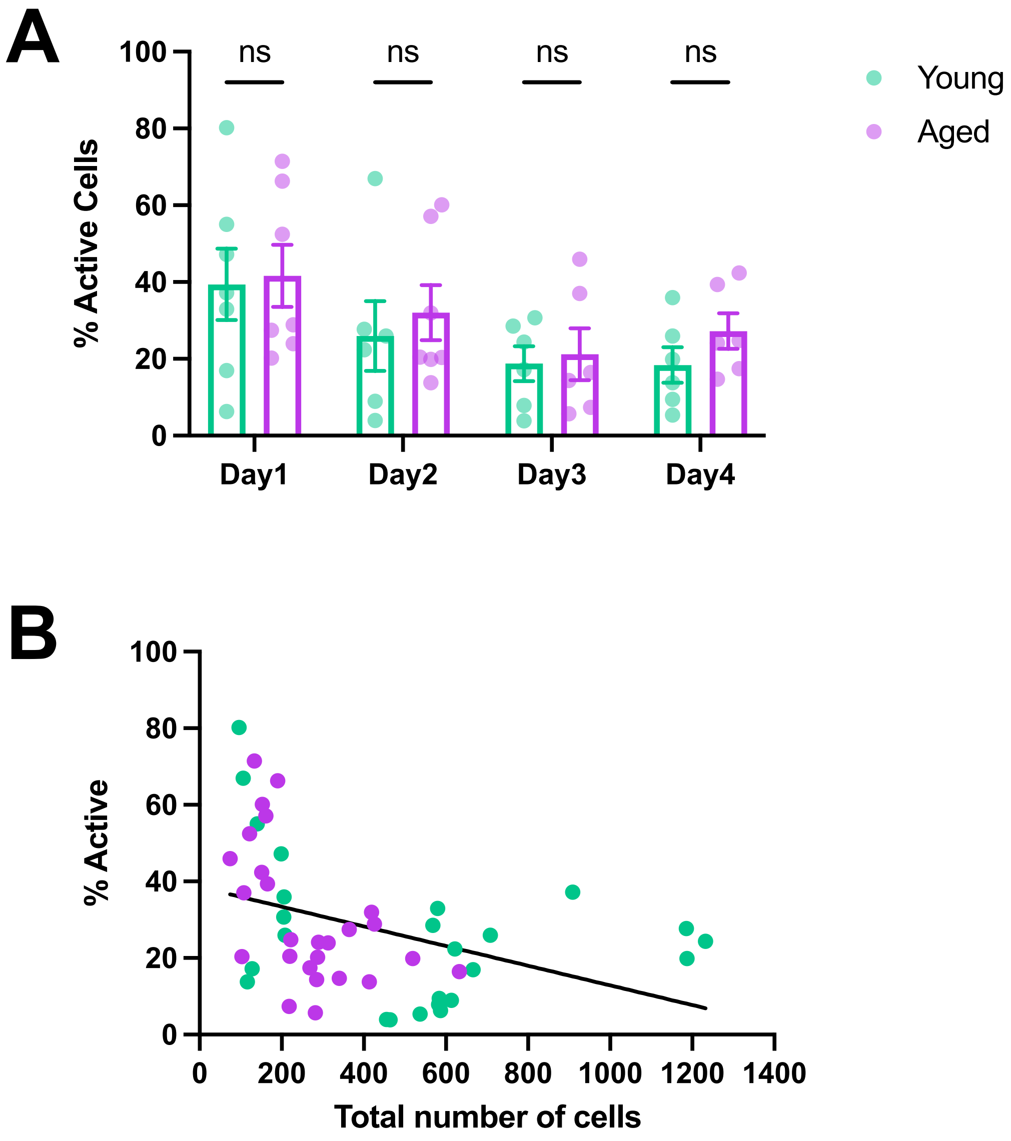

A
B
100
80
60
40
20
0
ns
ns
ns
ns
% Active Cells
Day1
Day2
Day3
Day4
Young
Aged
% Active
0
200
400
600
800
1000
1200
1400
Total number of cells

Figure S2

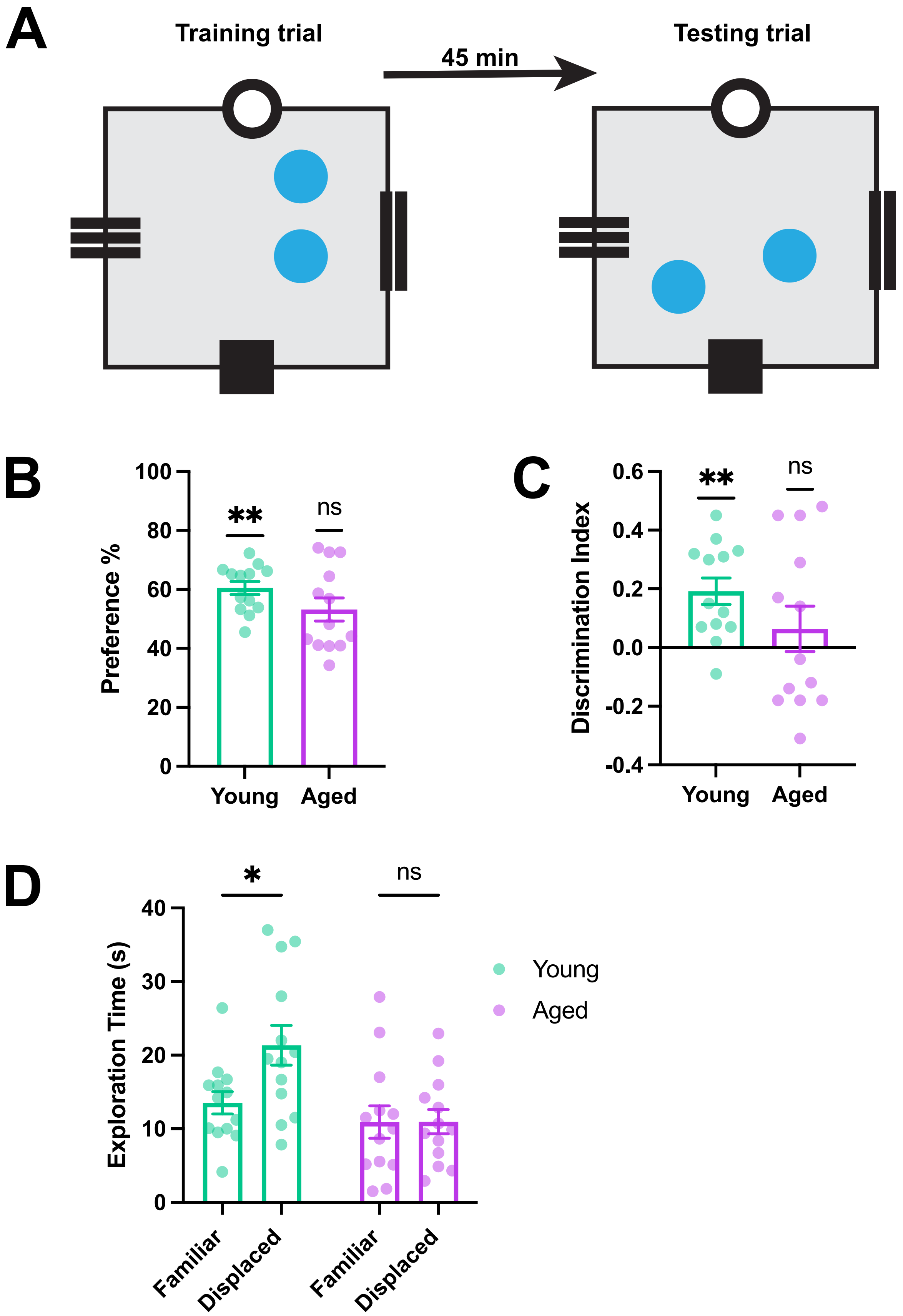
A
Training trial
45 min
Testing trial
B
Preference %
100
80
60
40
20
0
**
ns
Young
Aged
C
Discrimination Index
0.6
0.4
0.2
0.0
-0.2
-0.4
**
ns
Young
Aged
D
Exploration Time (s)
40
30
20
10
0
*
ns
Young
Aged
Familiar
Displaced
Familiar
Displaced

Figure S3

Supplementary Table 1.

Bregma-lambda distance	Anteroposterior	Mediolateral	Dorsoventral
3	1.5	1.5	1.8
3.2	1.6	1.55	1.8
3.4	1.7	1.6	1.9
3.6	1.8	1.65	1.9
3.8	1.9	1.7	1.95
4.0	2.0	1.75	2.0

Units: mm
