# Supplementary material for: Delayed formation of neural representations of space in aged mice
Source: Aging Cell. 2023 Jul 25;22(9):e13924. doi: 10.1111/acel.13924 (PMC10497831; doi:10.1111/acel.13924)
Supplement: Supplementary file 1 — Appendix S1. [file ACEL-22-e13924-s001.zip › TableS1_caption.docx]

**Table S1. Coordinates for viral injections into the dentate gyrus.** Coordinates are given as a function of the size of the skull, as measured by the distance between bregma and lambda (first column). The anteroposterior coordinate is the midpoint between bregma and lambda and the mediolateral coordinate is shown as a distance from the midline. The dorsoventral coordinate (depth of injection) is shown as a distance from the dura.
